# Striving for equity: An update from the *Journal of the Medical Library Association*

**DOI:** 10.5195/jmla.2021.1279

**Published:** 2021-07-01

**Authors:** Katherine G. Akers, Ellen M. Aaronson, Kathleen Amos, Kelsa Bartley, Alexander J. Carroll, Thane Chambers, John W. Cyrus, Erin R. B. Eldermire, Brenda Linares, Beverly Murphy, Melanie J. Norton, JJ Pionke, Amy Reyes

**Affiliations:** 1 katherine.akers@wayne.edu, Editor-in-Chief and Equity Workgroup Member; 2 ellen.aaronson@gmail.com, Resource Reviews Editor, MLA Proceedings Editor, and Equity Workgroup Member; 3 kamos@phf.org, Associate Editor; 4 k.bartley@med.miami.edu, Equity Workgroup Member and Liaison to MLA DEI Committee; 5 alexander.j.carroll@vanderbilt.edu, Assistant Editor; 6 thane@ualberta.ca, Assistant Editor and Equity Workgroup Member; 7 cyrusjw@vcu.edu, Editorial Board Member and Equity Workgroup Member; 8 erb29@cornell.edu, Editorial Board Member and Equity Workgroup Member; 9 blinares@kumc.edu, Equity Workgroup Member and Liaison to MLA Board of Directors; 10 beverly.murphy@duke.edu, Obituaries Editor, Equity Workgroup Member, and Liaison to MLA's African American Medical Librarians Alliance Caucus; 11 melanie.norton@yale.edu, Book Reviews Editor and Equity Workgroup Member; 12 pionke@illinois.edu, MLA Proceedings Editor and Equity Workgroup Member; 13 abreyes@library.ucla.edu, Equity Workgroup Member and Liaison to MLA's Latinx Caucus

## Abstract

In 2020, the *Journal of the Medical Library Association* (*JMLA*) launched an initiative aimed at providing more equitable opportunities for authors, reviewers, and editorial team members. This editorial provides an update on the steps we have taken thus far to empower authors, increase the diversity of our editorial team, and make equity-minded recommendations to the Medical Library Association.

In 2020, the *Journal of the Medical Library Association* (*JMLA*) launched an initiative aimed at providing more equitable opportunities for authors, reviewers, and editorial team members. As part of this initiative, *JMLA* editors formed an equity workgroup of select *JMLA* editors and editorial board members focused on implementing this work and issued a continuing call for manuscripts that address social injustices; speak to diversity, equity, and inclusion (DEI) in our workforce and among our user communities; share critical perspectives on health sciences librarianship; or are authored by individuals who are Black, Indigenous, and People of Color (BIPOC).

This work, however, was punctuated by mistakes made by the *JMLA* editor-in-chief and copyeditor, who did not honor the voices of five Black authors [1, 2, 3] invited to publish an editorial on anti-Blackness in librarianship [4]. This failure prompted *JMLA*'s editors and equity workgroup members to deepen and accelerate our interrogation of journal processes and policies to identify and remove barriers to publishing in, reviewing for, or serving as *JMLA* editorial team members [5]. To help guide this work, the equity workgroup was joined by representatives from the Medical Library Association (MLA)'s African American Medical Librarians Alliance (AAMLA) and Latinx Caucuses, DEI Committee, and Board of Directors. In addition, all editors and editorial board members have dedicated extra time and effort toward making *JMLA* a journal that better serves our stakeholders. So far, we have taken the following steps:

## EMPOWERING AUTHORS

Authors submitting to *JMLA* now have the option of recommending peer reviewers who are particularly well suited to comment on their manuscript. To reduce the power imbalance between editors and authors, editors now inform authors that they are not required to make every change recommended by a reviewer or accept edits that change the meaning of the text or alter ideas they wish to convey. Rather, authors are asked to justify their decision when they differ in opinion with a reviewer and to engage in dialogue with their editor. After acceptance, the copyeditor now sends authors a copyedited version of their manuscript showing tracked changes before the proof stage so they can more easily see and respond to the copyedits.

## INCREASING EDITORIAL TEAM DIVERSITY

A recent survey of *JMLA* editorial board members, reviewers, and authors showed that most are white, heterosexual, women, or without disabilities [6]. To increase the diversity of the editorial team, the editor-in-chief and equity workgroup recently sought and appointed colleagues from underrepresented groups to serve as an assistant editor, obituaries coeditors, and social media editor. Also, the editor-in-chief and editorial board redefined the function of editorial board members and, together with the equity workgroup, are implementing new, more inclusive strategies for recruiting editorial board members who are representative of a broader range of identity groups, professional roles and workplaces, and geographies. To better represent *JMLA*'s broad readership, we anticipate these editorial board members will include both MLA members and nonmembers as well as both health sciences librarians and individuals working in roles that support or are adjacent to health sciences librarianship.

## MAKING RECOMMENDATIONS TO MLA

The *JMLA* editorial board recommended that MLA adopt C4DISC's Joint Statement of Principles (https://c4disc.org/principles/) and appoint a taskforce to update the MLA Style Manual, particularly regarding the use of race- and ethnicity-related terms and the flexibility with which the manual should be applied in different situations.

## FUTURE STEPS

Our next steps will focus on providing continuing education to all *JMLA* team members to enrich our understanding of how systemic inequities impact scholarly publishing and editorial decisions. We will further bolster peer review by issuing a call for reviewers with expertise in DEI issues and critical theory, including information in our reviewer guidelines about recognizing and guarding against implicit bias, and exploring the possibility of holding an annual webinar for reviewers on topics related to DEI and bias. In addition, *JMLA* editors and editorial board members are planning to develop an editorial internship program to provide health sciences librarians who are new to scholarly publishing—particularly those from underrepresented groups—with mentored peer review training and an insider view of journal editing.

## CONCLUSION

The *JMLA* editorial team seeks to publish articles that address DEI issues among our health sciences library workforce and user communities, even—and especially—when they serve to disrupt the status quo or question core assumptions in our profession. Toward this end, we are constructing an editorial team that is more diverse, and better prepared, to handle manuscripts on a wider range of critical topics. We will continue to enact changes to *JMLA*'s process, policies, and programs to make engaging with *JMLA* a more welcoming and inclusive experience and commit to keeping our stakeholders informed of our progress. We also welcome your feedback and ideas for improvement, which can be sent to jmla@journals.pitt.edu or submitted through *JMLA*'s anonymous virtual suggestion box (https://www.mlanet.org/p/su/rd/survey=dd8fcbda-649f-11eb-8b4f-bc764e103913).
